# Migration of primordial germ cells and their relationship of PGCs with sex development in transgenic germline-specific fluorescent freshwater angelfish (*Pterophyllum scalare*)

**DOI:** 10.1038/s41598-025-85480-7

**Published:** 2025-01-08

**Authors:** Wai-Kwan Chu, Shih-Chin Huang, Ching-Fong Chang, Jen-Leih Wu, Hong-Yi Gong

**Affiliations:** 1 Marine Molecular Genetics & Biotechnology Laboratory, Department of Aquaculture, National Taiwan Ocean University, Keelung, 202301 Taiwan; 2 Center of Excellence for the Oceans, National Taiwan Ocean University, Keelung, 202301 Taiwan; 3 Institute of Cellular and Organismic Biology, Academia Sinica, Taipei, 11529 Taiwan; 4 Fisheries Research Institute, Kinmen County, 893 Taiwan; 5 College of Life Sciences, National Taiwan Ocean University, Keelung, 202301 Taiwan

**Keywords:** Freshwater angelfish, PGCs migration, Transgenic, Sex determination, Biotechnology, Cell biology, Developmental biology, Genetics

## Abstract

Primordial germ cells (PGCs), the progenitors of gametes, are essential for teleost reproduction. While their formation is conserved across teleosts, the activation, migration routes, and localization periods vary among species. In this study, we developed a novel transgenic line, *Tg(ddx4:TcCFP13-nanos3)*, based on the Nile tilapia genome, to label PGCs with clear fluorescent signals in the freshwater angelfish (*Pterophyllum scalare*). Our findings reveal a complex, multistage PGCs migration process in angelfish, with a significantly extended localization period (168 hpf) compared to zebrafish (24 hpf). Notably, individual differences in PGCs abundance were observed during early somite development. Analysis of PGCs counts and subsequent sexual maturation demonstrate a potential correlation between PGCs abundance and sex determination: 90% of PGCs-reduced individuals developed as males, while 83% of PGCs-rich individuals developed as females. This study provides a foundation for understanding PGCs migration and sex development in freshwater angelfish, offering valuable insights into reproductive biology and ornamental fish species. Furthermore, this in vivo PGCs tracking system for Cichlids provides a versatile tool for advancing research and applications in germ cell biology.

**Supplementary Information:**

The online version contains supplementary material available at 10.1038/s41598-025-85480-7.

## Introduction

The reproductive biology of teleost is a crucial field for advancing aquaculture development^1^. Understanding the mechanisms behind the formation of germ cells is imperative for sexual reproduction since it relies on the gametes produced by the germline. The sexual dimorphism of gametes plays a crucial role in transmitting genetic information and succeeding in generation. In various studies of model species such as zebrafish and medaka, researchers have clarified the regulations and mechanisms involved in primordial germ cells (PGCs), emphasizing the critical significance of understanding PGCs as they serve as precursors to gametes^2^. In teleost, PGCs are proven to form through preformation mechanisms and are specified during early embryogenesis^3^. Notably, they constitute a group of cells that uniquely engage in migration activity to reach the genital ridge. The accurate formation, migration, and localization of PGCs within this process are crucial for achieving normal reproduction^4^. Previous studies have employed diverse methodologies to identify molecular markers specific to PGCs and germ cells, enabling their distinction from other embryonic cells^5^. In addition to conventional immunofluorescence staining techniques, researchers commonly utilize the genetic lineage tracing approach, constructing reporter molecules with specific genes or elements that exclusively activate in PGCs. This approach allows researchers to thoroughly monitor and study the developmental path of PGCs in vivo.

To visualize the PGCs migration in living embryos, chimeric mRNA strategy and transgenic system were reported to be able to identify and track the PGCs in different teleosts. Numerous studies have reported that the 3′UTR region of those germ cells’ specific genes, such as *nanos3*, *dnd1,* and *ddx4 (vasa)*, were responsible for targeting the specific mRNA to the PGCs, thus critical for specifying the PGCs for identification, avoiding ubiquitous mRNA expression^6^. The chimeric mRNA strategy, conducting a fluorescent reporter gene fused with specific gene 3′UTR, has been widely used in labeling the PGCs. Various studies have approached this method succeeded in identifying PGCs in various species, such as medaka, Japanese flounder, red sea bream, turbot, and Atlantic salmon, achieve to label^7–11^. Although this method may generate the PGCs-labeled line in a shorter period, this expression pattern is not able to transmit along generations. The transgenic strategy is an essential tool for gene regulation studies, which provides stable foreign gene expression and is reproducible for mass-producing transgenic fish for further study needs^12^. Researchers efficiently isolate PGCs in rainbow trout by producing GFP-labeled PGCs through developing transgenic fish^13^. Numerous studies have successfully developed transgenic fish labeled with PGCs in zebrafish. In teleost, PGCs labeling across various species, including zebrafish, medaka, tilapia, and Prussian carp, has revealed a significant correlation between the number of PGCs or undifferentiated germ cells and sexual dimorphism in sex development^14–17^. Overall, the comprehensive exploration of PGCs across teleost species not only establishes a linkage between the abundance of these cells and sexual dimorphism in gonad development but also sheds light on various aspects, including the migration activity visualization, sexual development mechanisms, creation of sterile fish, and the exploration of gene regulation^18–22^.

The migration of PGCs during embryonic development constitutes a complex process regulated by diverse genetic signals and maintenance mechanisms. Although substantial progress has been achieved in comprehending this phenomenon in model organisms such as zebrafish and medaka, the biodiversity present in non-model animals introduces specific aspects that represent an intriguing frontier with potential differences yet to be fully explored. Freshwater angelfish, native to South America and belonging to the Cichlidae family, is one of the famous and popular ornamental freshwater fish species with many commercialized strains generated by selective breeding. The genetic information of freshwater angelfish remained unclear until the first study reported the whole genome sequencing and assembly of a pet angelfish after it died^23^. We established the transgenic technology by microinjection of one-cell fertilized eggs to generate the first transgenic pink angelfish line expressing *Acopora* coral red fluorescent protein in the muscle driven by zebrafish *ckmb* promoter/enhancer^24^. From F1 generation in 2013, the pink angelfish line was stably inherited to more than ten generations. Recently, we successfully applied CRISPR/Cas9 genome editing technology in freshwater angelfish to knockout the germ cells-specific *dnd1* gene, created a sterile angelfish model. Interestingly, we observed a male-biased sex development phenomenon exhibited in *dnd1* mutants^25^. Based on these findings, in addition to being essential for fertility in both male and female freshwater angelfish, we hypothesize that, PGCs may also influence sex differentiation during gonad development, as this species is gonochoristic. This inspired us to delve into uncovering the relationships between germ cells and sex development in freshwater angelfish. However, the lack of information regarding PGCs, sexual dimorphic development, and the regulatory mechanisms in sex determination of freshwater angelfish poses a primary challenge for us, hindering further exploration in freshwater angelfish. To address this issue, comprehending the behavior and role of PGCs in freshwater angelfish becomes critical.

In this study, we aimed to label PGCs to uncover their migration route and localization position, providing crucial information about PGCs in freshwater angelfish by generation of a heritable PGC labeling line through a transgenic strategy. According to research in PGCs, the *ddx4*/*vasa* gene emerged as a comprehensively studied gene, which is specifically expressed in germ cells and highly conserved among species during evolution. In zebrafish, studies have reported that fragments of 8.5 kb or 8.3 kb upstream of the *ddx4* gene can recapture the expression pattern of a reporter gene to mimic endogenous *ddx4* expression^26–29^. Notably, a 2.3 kb 3′ flanking fragment of *ddx4* gene provides specificity in labeling PGCs and developmental germ cells in zebrafish. In medaka, researchers successfully composed an expression vector using a 5.1 kb promoter fragment along with a 0.64 kb 3′ region of the medaka *ddx4* gene, enabling the labeling of PGCs and developmental germ cells^30^. We were inspired by studies achieving a comprehensive lifetime tracking of germ cells in zebrafish, in which the *piwil1* promoter was combined with a *nanos3* 3′UTR along with early post-transcriptional regulation elements and later transcriptional regulation sequences, to visualize the entire lifetime of germ cell development, from PGCs to oocytes in females and spermatids in males^19^. By utilizing a combination of germ cell-specific regulation element fragments, we may not only achieve label PGCs but also cover the entire lifetime of germ cell development in freshwater angelfish in our study. According to the latest research in Nile tilapia, a comprehensive evaluation study revealed that *nanos3*, *dnd1*, *piwil1*, and *ddx4* gene expression patterns would be viable for specifically targeting PGCs and germ cells in tilapia^31^. Depletion of *nanos3* has been reported to lead to sterility in Nile tilapia, indicating these are critical genes for maintaining germ cells in tilapia^17^. As stated above, based on the studies in zebrafish and Nile tilapia, we designed an expression construct referencing the genome sequence of Nile tilapia, a species in the same Cichlidae family as freshwater angelfish. This construct combined the *ddx4* promoter and *nanos3* 3′UTR to create the expression vector to achieve the labeling of PGCs and germ cells in freshwater angelfish. Importantly, we successfully generated the transgenic freshwater angelfish line, *Tg(ddx4:TcCFP13-nanos3)*, demonstrating the lifetime labeling of PGCs and developmental germ cells in freshwater angelfish. Our findings revealed that PGCs abundance varies among freshwater angelfish larvae and is correlated with sex dimorphisms in their development.

## Materials and methods

### Approval for animal experiments

The animal use protocol for this study was reviewed and approved by the Institutional Animal Care and Use Committee (IACUC) of the National Taiwan Ocean University. The IACUC Approval No. is 111074 pertains to freshwater angelfish, while IACUC Approval No. is 108042 pertains to zebrafish. All experiments were conducted using the best practices to minimize animal suffering and followed the guidelines of IACUC. Also, all animal experiments conducted in this study are in accordance with the ARRIVE guidelines (https://arriveguidelines.org).

### Maintenance of experimental animal

The experimental animals used in this study included Zebra freshwater angelfish (*Pterophyllum scalare*) and wild-type zebrafish (AB strain). The freshwater angelfish were sourced from the commercial ornamental fish company Jy Lin Trading Co. Ltd (Pingtung, Taiwan). The fish were housed in indoor tanks with a consistent temperature of 28.5 °C and a photoperiod cycle of 14 h of light and 10 h of darkness. They were fed a diet of Tetra Bits Complete (Tetra, Melle, Germany) and artemia, with feeding schedules adjusted based on developmental stages to ensure optimal nutrition. For breeding, male and female angelfish were paired in a 1:1 ratio and placed in a separate tank with a spawning slate for egg-laying.

Wild-type zebrafish (AB strain) were maintained in an indoor recirculating system at 28 °C under the same photoperiod conditions as the angelfish. Regular care of zebrafish followed established protocols, including feeding and maintenance practices to ensure optimal health and growth^32^.

### Construction of expression vector and transgenic fish line generation

A transgenic expression construct was designed to trace PGCs migration based on the genome sequence of the *ddx4* gene in Nile tilapia (*Oreochromis niloticus*), referenced from NCBI (Gene ID: 100698352). A 4426 bp transcriptional regulatory element, including the 5′ UTR and promoter region of the *ddx4* gene (GenBank accession number: PQ031198), was analyzed using Genomatix Software (www.genomatix.de) to predict potential transcription factor binding sites (TFBS) with a threshold of 0.95 for the position weight matrix (PWM) to ensure high accuracy in the identified sites. The fragments were amplified with designed primers. The primers contained a restriction enzyme site with XhoI and StuI. The forward primer is 5′-CTCGAGTTATATCTTCTGACATGTTTGGAGTTTTGATTTGTTTC-3′, and the reverse primer is 5′-AGGCCTTCTTGCTCACCTGAGGAAAAAAGTACCAC- 3′. The 3′UTR (GenBank accession number: PQ031199) of the transgenic expression construct involved a fragment of the *nanos3* gene in Nile tilapia (Gene ID: 100698891), specifically chosen for providing PGCs-specific labeling ability. The fragment included the 3′UTR and 3′ flanking region of the *nanos3* gene and contained a restriction enzyme site with NheI and KpnI. The forward primer is 5′-GCTAGCACCAGCAGGTGGCAAGGAGCAATAAGACACTAC-3′, and the reverse primer is 5′-GGTACCAACTCCGCTCGTTAAGAGAGTTATTTCAGGTTTC-3′. According to the manufacturer’s protocol, these fragments were amplified using Q5® High-Fidelity DNA Polymerase (NEB, Ipswich, USA). After purification, the fragments were ligated into the pGEM®-T Easy vector for sequencing. Following digestion from pGEM®-T Easy vectors using specific restriction enzymes, the fragments were cloned separately into the pT2 plasmid modified from pT2KXIG plasmid (a kind gift with pCS-TP plasmid from Dr. Koichi Kawakami in National Institute of Genetics, Japan)^33^. The 4426 bp *ddx4* 5′-regulatory fragment was inserted upstream of the coding sequence of the reporter fluorescent protein *TcCFP13* of the pT2 construct. The *TcCFP13*, a cyan fluorescent protein utilized as a reporter gene in this study, was sourced from Taiwan coral (*Acropora* sp.) provided by Dr. Ming-Chyuan Chen (National Kaohsiung University of Science and Technology, Kaohsiung, Taiwan)^34^. Replacing the SV40 (Simian virus 40) Poly A in the 3′UTR region of the pT2 plasmid with the *nanos3* 3′UTR combined with the 3′ flanking fragment, generated the transgenic plasmid, pT2*-ddx4-TcCFP13-nanos3*.

Given the long generation time of freshwater angelfish, zebrafish were selected as a candidate species to efficiently evaluate the molecular construct’s specificity. The transgenic lines of both zebrafish and freshwater angelfish were generated via microinjection. For zebrafish, the injection solution was prepared as follows: 50 ng/μl of transgenic vector pT2-*ddx4-TcCFP13-nanos3* plasmid DNA, 0.1 M KCl, 5% phenol red (used as a dye), and 50 ng/μl *Tol2* transposase mRNA prepared from pCS-TP by in vitro transcription^33^. Breeding procedure was conducted as the following the protocol^32^. Fertilized eggs were collected, and a 1–4 nl of mixed microinjection solution was injected into 1-cell stage embryos. The F0 generation of zebrafish was raised until three months old to reach sexual maturity. Crossbred them with wildtype zebrafish and screened out the embryos with positive fluorescent signals to obtain the F1 generation and, subsequently, F2 generations.

For freshwater angelfish, we collected the spawned eggs from the spawning slate and ensured their fertilization under a stereomicroscope. The injection solution was prepared with 50 ng/μl of pT2-*ddx4-TcCFP13-nanos3* plasmid DNA, 0.1 M KCl, 8% phenol red, and 50 ng/μl *Tol2* transposase mRNA. Gently mixed and injected 1–3 nl into the animal pole of the 1-cell embryos of freshwater angelfish. The injected embryos were collected and maintained in a petri dish, and typically developing individuals were selected to generate the F0 generation. F0 individuals were raised until they reached sexual maturity and were then cross-mated with wild-type freshwater angelfish. Transgenic individuals were identified using fluorescence stereomicroscopy, enabling the selection of potential transgenic founders. Founders able to transmit the transgene to the F1 offspring were selected, and they were crossbred with wild-type individuals to generate F1 and, subsequently, F2 generations. Offspring with positive fluorescent signals were screened from each generation using Leica Z16 APO fluorescent stereomicroscopy (Leica, Wetzlar, Germany). Stability across generations was assessed by selecting lines exhibiting stable and heritable transgene expression, with observations and imaging records being recorded.

### Definition of embryonic stages and imaging processing of PGCs migration

The morphology of embryonic development and the process of PGCs migration in both zebrafish and freshwater angelfish were observed using Leica Z16 APO fluorescent stereomicroscopy (Leica) combined with the white light LED illumination system pE-300 lite (CoolLED, Andover, United Kingdom). Developmental stages were defined based on established studies in zebrafish and freshwater angelfish^35,36^. Fluorescence imaging was captured using the K5 sCMOS Microscope Camera (Leica).

Fertilized eggs were collected from the breeding tanks and transferred to a petri dish incubated at 28 °C. Observations were conducted at specific embryonic developmental stages. To evaluate the specificity of the molecular construct and trace PGCs migration activity, transgenic fish with positive fluorescence signals were screened at early embryogenesis stages. 40 transgenic zebrafish embryos and 168 transgenic freshwater angelfish embryos were observed over time to monitor the entire process of PGCs migration. A schematic representation of the novel PGCs migration pattern in freshwater angelfish was generated based on these observations. Representative images of PGCs migration in freshwater angelfish were recorded from a single embryo to ensure accuracy. These were included in: Fig. 2 and Fig. 3 (256-cell to high stage), Fig. 3 (dome stage to 7 days after hatching), Fig. 6 (PGCs-rich), and Fig. 6 (PGCs-reduced). These images provide comprehensive documentation of the migration patterns and routes.

To analyze PGCs abundance variation, offspring were obtained from a single pair of inbred F1 freshwater angelfish. Counts were conducted at the larva stage, 1 day after hatching. Independent breeding events were carried out to carefully screen and identify ‘PGCs-reduced’ and ‘PGCs-rich’ larvae, bred from the same pair of F1 parents. Multiple layered photographs of the larvae from both lateral views were captured to assess PGCs numbers. These two groups were raised separately, and sex identification was performed upon reaching sexual maturity.

### Histological analysis of freshwater angelfish PGCs

Larvae were collected at 1, 5, and 7 days after hatching and fixed in 7% formalin for 48 h. Following fixation, the larvae were transferred to 70% ethanol for 24 h, then dehydrated through a graded ethanol series (80%, 90%, and 100%). The samples were cleared in xylene and embedded in paraffin wax. Once the molten wax solidified in molds, specimen blocks were sectioned into 6 µm-thick slices using a HistoCore MULTICUT Microtome (Leica Biosystems, Nußloch, Germany). The sections were stained with hematoxylin and eosin and observed under a light microscope for histological analysis.

## Results

### Establishment of a germline-specific transgenic vector by referencing the genome of Nile tilapia

**Fig. 1:**
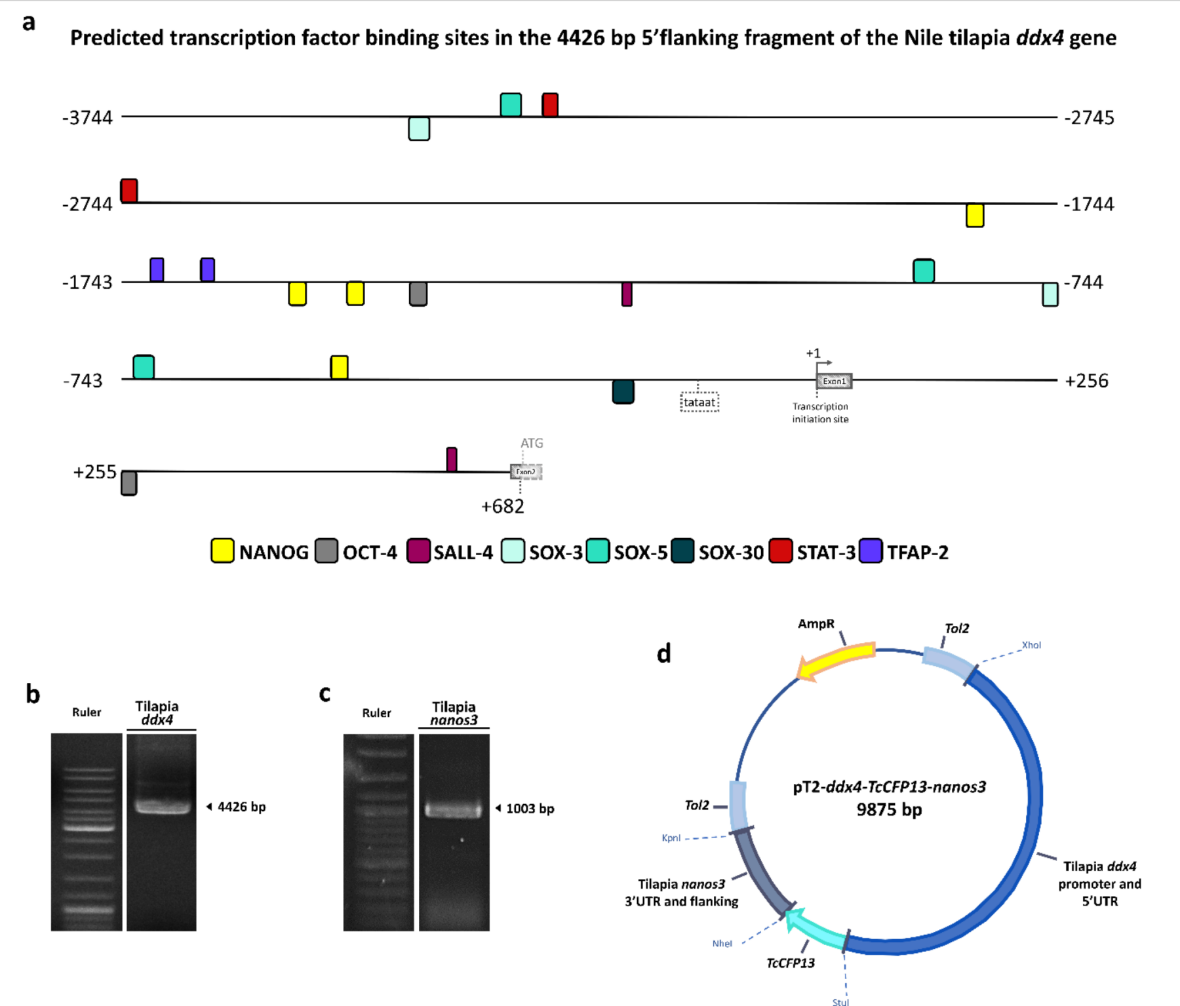
Establishment of a germline-specific transgenic expression construct for PGCs labeling. (**a**) Bioinformatics prediction of the potential transcription factor binding sites (TFBS) within a 4426 bp 5′ regulatory fragment of Nile tilapia *ddx4* gene. It includes 3744 bp promoter, exon 1, intron 1, and up to the translation initiation codon (ATG) in exon 2, based on NCBI Gene ID: 100698352. Different colors indicate specific potential TFBS of NANOG, OCT-4, SALL-4, SOX-3, SOX-5, SOX-30, STAT-3, and TFAP-2. The transcription initiation site is designated as + 1, with upstream nucleotides numbered negatively. (**b**) Gel electrophoresis analysis of a 4426 bp fragment amplified from the genome of Nile tilapia, including the promoter and 5′UTR region of the *ddx4* gene. Bands are cropped from different sections of the same gel; original gels are presented in Supplementary Fig. 1. (**c**) Gel electrophoresis analysis of a 1 kb fragment amplified from Nile tilapia, including the 3′UTR and flanking region of the *nanos3* gene. Bands are cropped from different sections of the same gel; original gels are presented in Supplementary Fig. 2. (**d**) Schematic illustration of the transgenic expression construct pT2*-ddx4-TcCFP13-nanos3*.

In this investigation, our primary goal was to label PGCs in vivo and unravel their migration dynamics, route, and localization patterns within the genital ridge of freshwater angelfish. Utilizing the *Tol2* transposon system, we established a transgenic line characterized by inheritable expression patterns, ensuring stability for subsequent experiments. As mentioned earlier, Nile tilapia, belonging to the Cichlidae family, was a reference for germ cell-specific expression genes. We designed an expression vector by incorporating Nile tilapia *ddx4* gene 4426 bp 5′-regulatory fragment and *nanos3* gene 1003 bp 3′-regulatory fragment to form the transgenic expression construct. In this study, we used a 4426 bp 5′-regulatory sequence, including the 3744 bp promoter region, exon 1, intron 1 and exon 2 sequences upstream of the start codon (ATG) of the Nile tilapia *ddx4* gene, for promoter analysis. Based on the results, potential transcription factor binding sites (TFBS) were predicted, and we identified several transcription factors (TF) that may play significant roles in reproduction or gene regulation in germ cells. The bioinformatics analysis using Genomatix revealed that several TFBS, such as NANOG, OCT-4, SALL-4, SOX-3, SOX-5, SOX-30, STAT-3, and TFAP-2, were identified within the 4426 bp fragment (Fig. 1a). Notably, four potential NANOG binding sites, which is a transcription factor reported to have a critical role in PGCs specification, were located within the distal region (− 520/− 502 bp, − 1503/− 1485 bp, − 1565/− 1547 bp, and − 1841/− 1823 bp) of the upstream fragment (Fig. 1a). Among the results, the TF included OCT-4, SALL-4, SOX-3, SOX-5, SOX-30, STAT-3, and TFAP-2, correlated with germ cells. Previous studies have identified that NANOG is a critical transcription factor during early germ cell development, involved in regulating the formation and specification of PGCs and early germ cell maintenance^37–40^. OCT-4 has been discovered to be expressed in PGCs and mature gonads^41^. SALL-4, SOX-3, SOX-5, SOX-30, STAT-3, and TFAP-2 also play roles in maintaining or regulating genes correlated with germ cells, highlighting their important functions in germ cell development^42–47^. These categorized TFBS were presumptively located between the -3437/ + 614 bp region (Fig. 1a). Based on the genomic database from Nile tilapia, we employed PCR to amplify and clone a 4426 bp fragment of the 5′ flanking and 5′UTR region of the tilapia *ddx4* gene and a 1 kb fragment downstream of the stop codon, containing the 3′UTR and 3′ flanking region of the tilapia *nanos3* gene (Fig. 1b,c). Sequencing confirmed no mutations in the colonies. Enzyme digestion and ligation were then employed to integrate these fragments into the recombinant plasmid, constructing an expression construct driven by the expression of the cyan fluorescent protein signal by the *ddx4* promoter, where the 3′ fragments of *nanos3* provided stability of the fluorescent signal and labeling ability of PGCs mRNA. The transgenic expression vector, pT2-*ddx4*-*TcCFP13*-*nanos3*, was successfully generated in this study (Fig. 1d).

### Evaluation of cross-species efficiency of PGCs labeling using zebrafish

Recognizing the relatively long generation time of freshwater angelfish, we initially used zebrafish to evaluate the efficiency and specificity of the expression construct in labeling PGCs. To this end, we generated the transgenic zebrafish line *Tg(ddx4:TcCFP13-nanos3)*. Derived from the tilapia genome, the construct robustly induces fluorescent protein expression and effectively labels PGCs during zebrafish embryonic development. These results underscore the construct’s potential as a cross-species tool for PGCs studies.

During the early embryonic stages, spanning from the sphere stage to gastrulation, faint background fluorescence was observed in the animal pole (Supplementary Fig. 3a,b). Specific cell signals were not discernible during this phase. However, during the segmentation period, specific fluorescent signals corresponding to individual cells became apparent, with labeled cells localizing bilaterally along the dorsal region. (Supplementary Fig. 3c). These signals were consistent with previous studies describing PGCs localization and migration patterns in zebrafish^48,49^. Throughout embryogenesis, PGCs exhibited dorsal migration surrounding the yolk sac and advancing towards the tailbud (Supplementary Fig. 3d). By the prim-5 stage, the migration was completed, and PGCs were localized at the genital ridge, corroborating prior findings on zebrafish PGCs migration (Supplementary Fig. 3e)^50,51^. These findings confirm that our expression construct not only successfully labels PGCs in zebrafish but also demonstrates its specificity and efficiency.

### Comprehensive characterization of multistage PGCs migration in freshwater angelfish

Building on the construct’s successful application in zebrafish, we expanded its use to freshwater angelfish to explore the developmental intricacies and migration patterns of PGCs. Embryos exhibiting positive and distinct fluorescent signals were meticulously selected for observation. Monitoring the entire embryonic development process in angelfish, we successfully labeled PGCs and traced their migration routes, uncovering a complex, multistage migration pattern unique to this species. PGCs migration of freshwater angelfish is highly synchronized among individuals within the same developmental timeline.

**Fig. 2:**
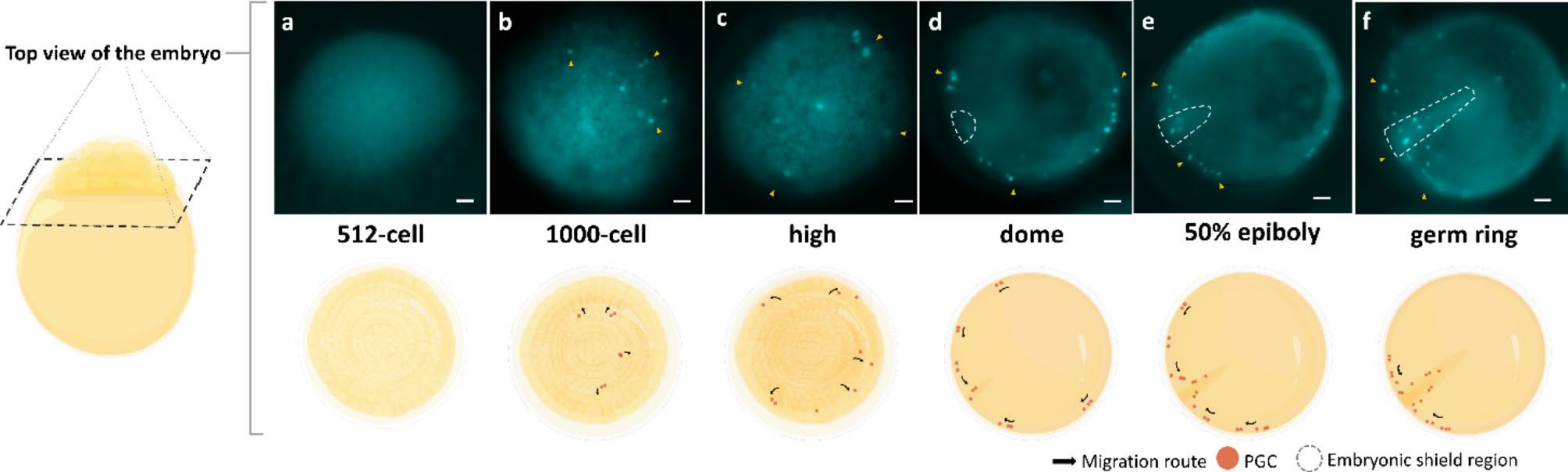
Micrograph visualizing PGCs migration from an animal pole (top) view during early embryonic development in *Tg*(*ddx4:TcCFP13*-*nanos3*) transgenic freshwater angelfish. No fluorescent signals were observed from the zygotic period through the 512-cell stage. Cyan fluorescent signals appeared in specific cells at the 1000-cell stage of embryonic development in freshwater angelfish. Yellow arrows indicate the PGCs, while white dotted lines mark the embryonic shield development region. Micrographs correspond to the following embryonic stages, including (**a**) 512-cell stage, (**b**) 1000-cell stage, (**c**) high stage, (**d**) dome stage, (**e**) 50% epiboly, (**f**) germ ring stage. Schematic illustrations depict PGCs migration for each corresponding embryonic stage. Bar: 100 μm.

**Fig. 3:**
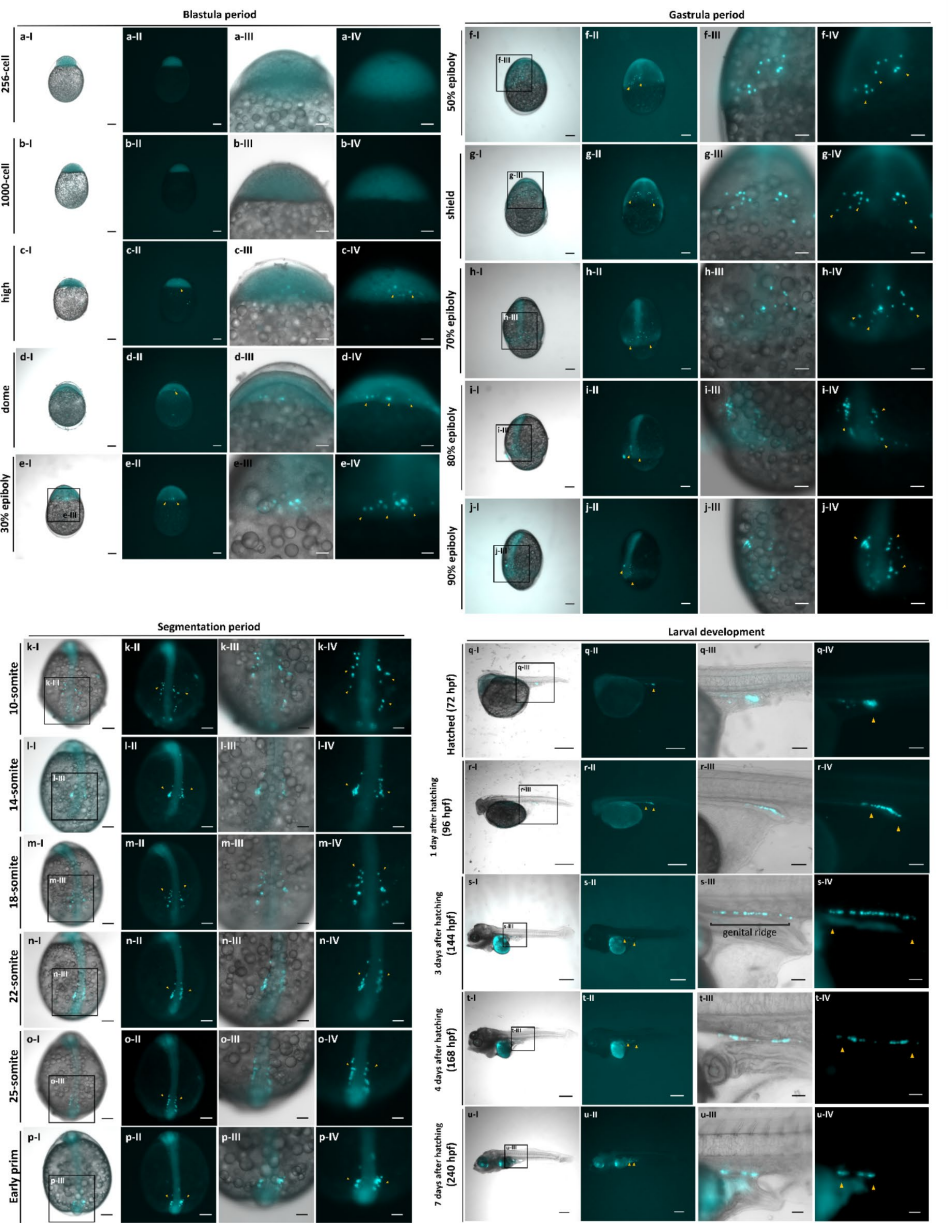
Micrograph visualizing the complete PGCs migration in *Tg*(*ddx4:TcCFP13*-*nanos3*) transgenic freshwater angelfish. Detailed migration routes and behaviors of PGCs were successfully tracked from the embryonic stages to the larva stage. Yellow arrows indicate PGCs. Micrographs illustrate the following views and periods of embryonic development, including (**a**–**e**) lateral view during blastula period, (**f**–**j**) dorsal view during gastrula period, (**k**–**p**) dorsal view during segmentation period, (**q**–**u**) side view during larva development. Bar: 500μm (**q**–**u**) I-II, 200μm (**a**–**p**) I–II, 100μm (**a**–**u**) III–IV.

Following fertilization, maternally derived positive fluorescent signals were observed in the animal pole during the zygote period, persisting through the cleavage period within blastomeres. However, specific cell signals within blastomeres were not discernible within the zygote and cleavage period (Supplementary Fig. 4; Figs. 2a, 3a). As embryonic development progressed, fluorescently labeled cells appeared randomly within the blastoderm (Figs. 2b,c; 3b,c). During the dome stage, these cells migrate toward the blastodisc margin, an indication of PGCs identity consistent with previous studies on germ cell behavior in teleost (Figs. 2d, 3d)^30,49,52^. Tracking PGCs migration from the animal pole revealed a conserved phenomenon during early epiboly: as the blastoderm expanded, PGCs labeled cells exhibited independent clockwise and anticlockwise migration patterns. PGCs continued migrating toward the embryonic shield development region (Figs. 2d–f, 3d–h). As the dorsal portion of embryos became distinctly thicker during the late gastrula period, PGCs continuously engaged in migration and converged near the dorsal region, congregating beside the dorsal development region during the late gastrula period at the 80% epiboly stage (Fig. 3i). When the blastoderm covered 90% of the yolk, PGCs had migrated towards the ventral side of the embryo, positioning themselves beside the dorsal region of yolk margin (Fig. 3j). During early somite development in the segmentation period, PGCs migrate straight toward the middle position between the two sides of the dorsal, and this migration process happens within the trunk-tail region of the freshwater angelfish embryo (Fig. 3j,k). Interestingly, upon initially reaching the middle, some PGCs halted migration, maintaining a stationary position until all PGCs occupied that location (Fig. 3l). Subsequently, at the 14-somite stage, PGCs forming in two cell clusters and resumed the migration dorsally toward the tail bud within the mesoderm (Fig. 3l–p). This phase was characterized by a more disciplined and synchronized migration activity, with cell clusters forming and migrating towards the dorsal mesentery. After hatching, PGCs were found to be located near the anus (Fig. 3q). Surprisingly, during larva development, PGCs continued to migrate predominantly in a straight path, eventually localizing within the genital ridge above the intestine in a linear arrangement (Fig. 3r,s). Fluorescent signals of PGCs persist up to 4 days after hatching, with no further migration activity observed (Fig. 3t). Signal depletion was noted by 7 days after hatching (Fig. 3u).

**Fig. 4:**
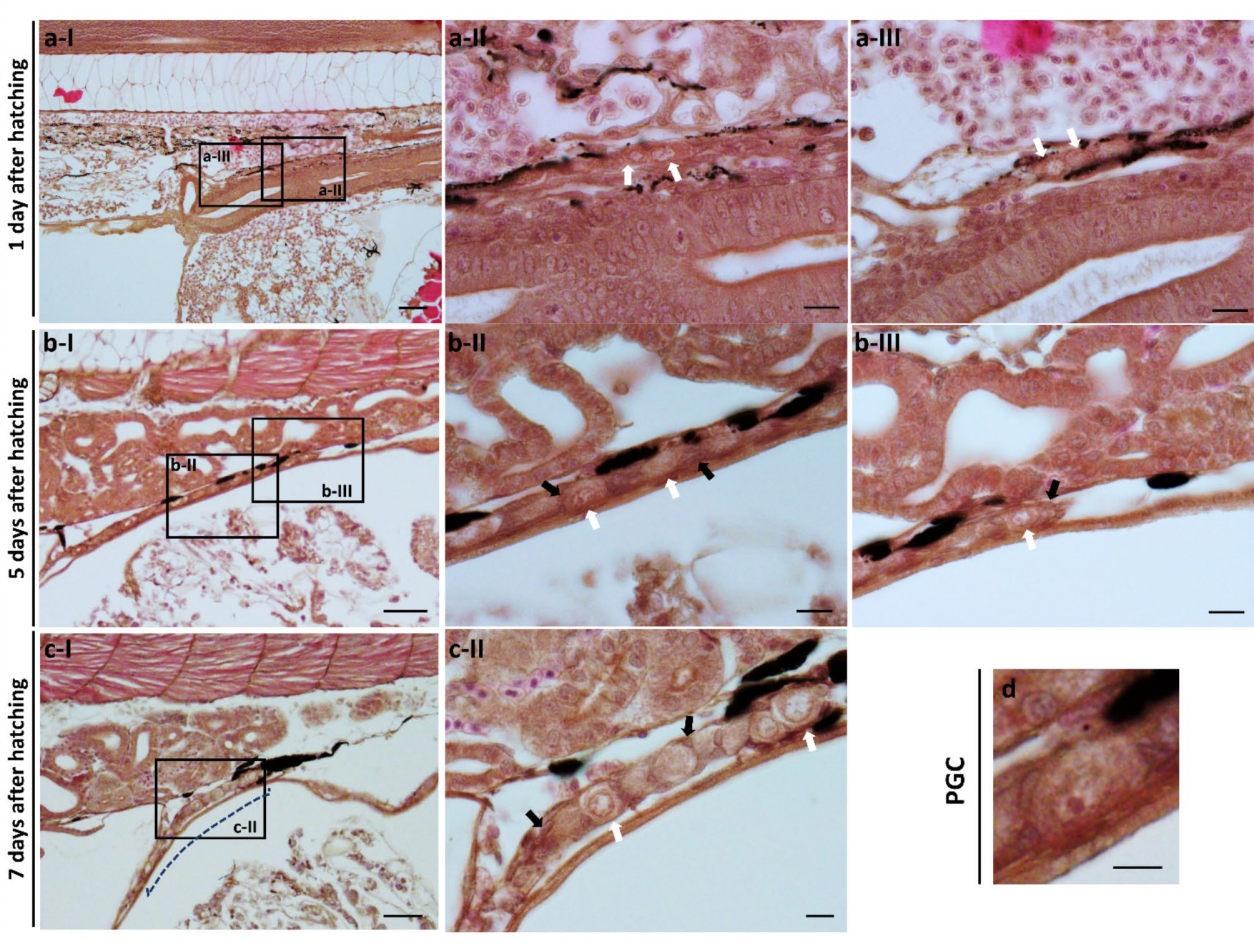
Histological analysis of PGCs in freshwater angelfish larvae at 1, 5, and 7 days after hatching. (**a**) Histological section of a larva at 1 day after hatching, showing PGCs. White arrows indicate PGCs. (**b**) Histological section of a larva 5 days after hatching, showing PGCs surrounded by somatic cells (stromal cells), forming a gonad. White arrows indicate PGCs; black arrows indicate somatic cells. (**c**) Histological section of a larva 7 days after hatching, showing undifferentiated gonad tissue with PGCs. The blue dotted line indicates the undifferentiated gonad; white arrows indicate PGCs; black arrows indicate somatic cells. (d) An individual PGC cropped from section (b-II), highlighting the size of the PGC. Bar: 100 μm (**a**) I, 50 μm (**b**, **c**) I, 10 μm (**a**, **b**) II–III and (**c**) II, 5 μm (**d**).

Additionally, we identified the PGCs of freshwater angelfish through histological sections to determine their characteristics. PGCs were located at the bottom of the somite at 1 day after hatching (Fig. 4a). At 5 days after hatching, histological sections showed a linear tissue located below the kidney and above the intestine, containing PGCs surrounded by somatic (stromal) cells, indicating the formation of the gonad at this stage (Fig. 4b). The undifferentiated gonad was identified in the same position at 7 days after hatching (Fig. 4c). This timeline suggests that PGCs may complete their migration and become localized at the genital ridge approximately 4 days after hatching. Moreover, this is the first identification of PGCs in freshwater angelfish through histological analysis, and their characteristics are consistent with previous descriptions of PGCs in teleosts (Fig. 4d)^53–56^.

**Fig. 5:**
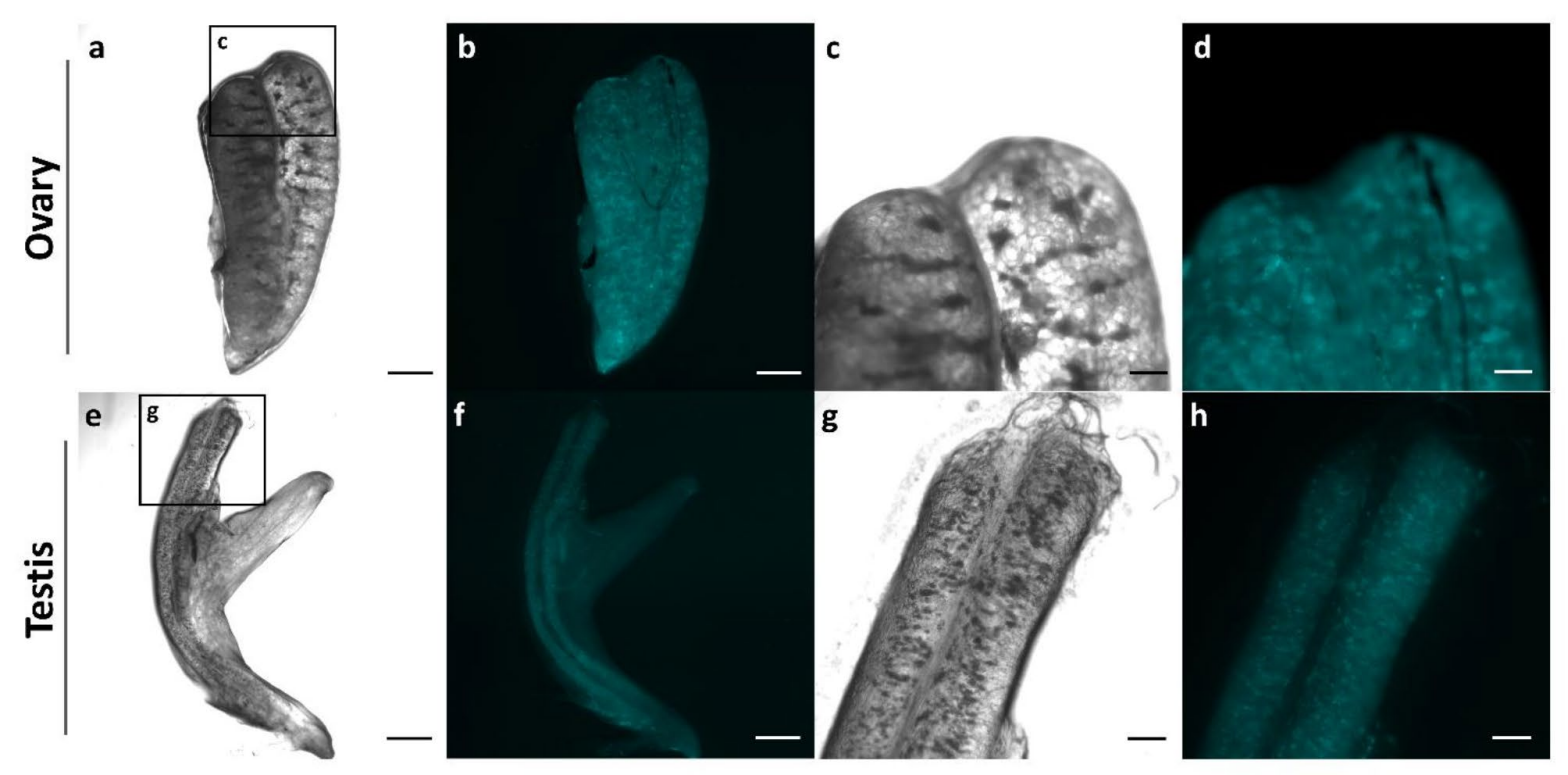
Micrograph visualizing the differentiated gonads of juvenile *Tg*(*ddx4:TcCFP13*-*nanos3*) transgenic freshwater angelfish. Gonads were dissected from juvenile fish, revealing positive cyan fluorescent signals within the differentiated gonads. The cell-like expression pattern indicates that germ cells were successfully labeled. Bar: 1000 μm (**a**, **b**, **e**, **f**), 200μm (**c**, **d**, **g**, **h**).

To further explore the versatility of this expression construct, we examined the differentiated gonadal tissue in juvenile freshwater angelfish at 4 months post-fertilization. Fluorescent signals were observed in developing ovaries and testes, demonstrating that the construct is capable of labeling not only PGCs but also differentiated germ cells within both ovarian and testicular fates (Fig. 5). This result reinforces the utility of this expression construct as a versatile tool for studying germ cell development in freshwater angelfish and potentially other Cichlids.

### PGCs abundance and its potential role in sexual dimorphism of freshwater angelfish

**Fig. 6:**
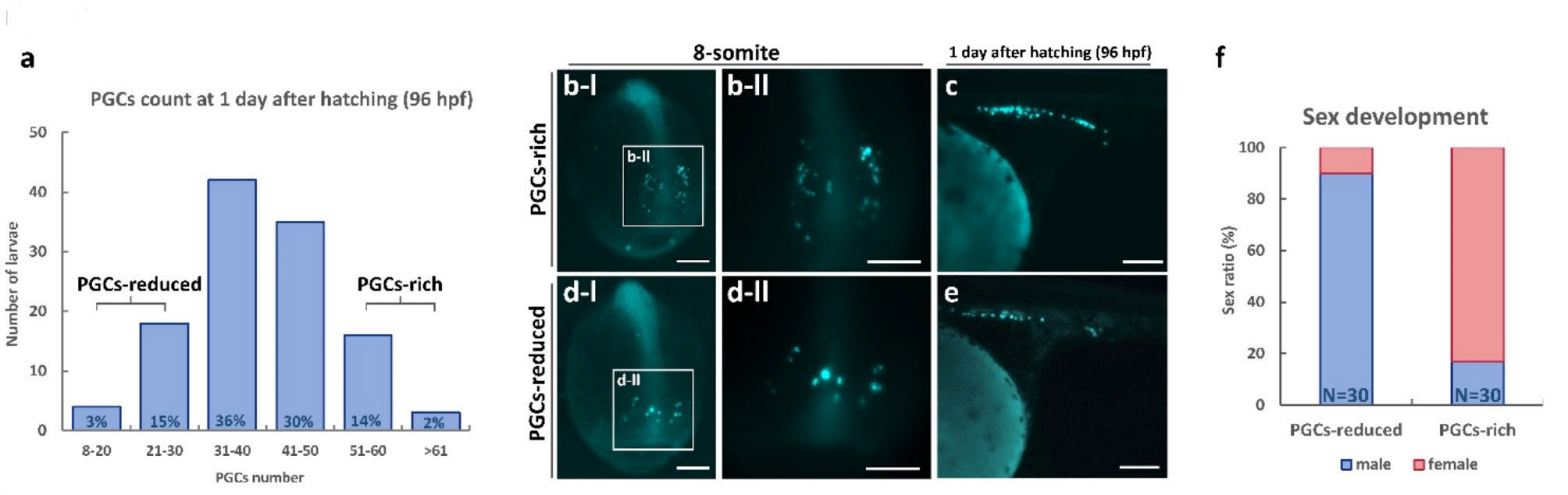
Analysis of PGCs abundance and its correlation with sexual development analysis in transgenic freshwater angelfish. Revealing the relationship between PGCs abundance and sexual development in freshwater angelfish. (**a**) Distribution of PGCs counts in embryos at 1 day after hatching (96 hpf). Variability in PGCs abundance among individuals is evident. Embryos with fewer than 31 PGCs (ranging from 8 to 30) were categorized as the PGCs-reduced group, while those with more than 50 PGCs were categorized as the PGCs-rich group. Intermediate groups (31–50 PGCs) are also presented for context. (**b**, **c**) Micrographs of the embryo from the PGCs-rich group showing higher PGCs abundance. (**d**–**e**) Micrographs of the embryo from PGCs-reduced group showing lower PGCs abundance. (**f**) Sex ratio analysis of PGCs-reduced and PGCs-rich groups, conducted after individuals reached sexual maturity. Larvae were carefully screened from a subsequent breeding event using offspring of the same parental pair to ensure consistency. A male-biased sex ratio was observed in the PGCs-reduced group, while a female-biased ratio was evident in the PGCs-rich group. Bar: 200 μm (**b**–**e**).

In our investigation of freshwater angelfish embryos, we observed notable variation in PGCs abundance since early somite development. Similar phenomena, where PGCs abundance is linked to sex development, have been reported in other teleost species, prompting us to investigate whether a similar relationship exists in freshwater angelfish^14,15,17,19,57^. To explore this, we collect embryos by inbred F1 transgenic parents and accurately count PGCs number at the late migration stage, one day after hatching (96 hpf). Among 118 larvae analyzed, most individuals had 31–50 PGCs, while a subset exhibited significant variations, with 18% possessing fewer than 31 PGCs (ranging from 8 to 30) and 16% having more than 50 PGCs (Fig. 6a).

Building on previous findings that infertile freshwater angelfish exhibit a male-bias sex development, contrasting with the 1:1 sex ratio observed in wildtype freshwater angelfish, we hypothesized that PGCs abundance may influence sexual dimorphism^25^. To investigate this, we focused on two extremer populations: individuals who had a low amount of PGCs (ranging from 8 to 30), defined as PGCs-reduced, and individuals who had a high amount of PGCs (more than 50), defined as PGCs-rich (Fig. 6a–e). We, therefore, carefully screened out these larvae by collecting the offspring inbred from the same pair of F1 parents, and the offspring from these groups were separated, with 30 individuals from each group raised to sexual maturity. Sex identification revealed an imbalance in sexual dimorphism: 27 out of 30 (90%) individuals in the PGCs-reduced group developed as males, while 25 out of 30 (83%) individuals in the PGCs-rich group developed as females (Fig. 6f). These findings indicate a potential link between PGCs abundance and sex development in freshwater angelfish.

This observation aligns with reports from other teleost species, where variations in germ cells abundance have been implicated in influencing sex development pathways. Future studies should aim to elucidate the mechanisms underlying this phenomenon in freshwater angelfish, particularly whether PGCs abundance directly regulates gonadal differentiation or interacts with other factors influencing sex determination. This work highlights the potential role of PGCs abundance in shaping sexual dimorphism, contributing to our understanding of germ cells biology in teleost.

## Discussion

Our study presents a pioneering approach to visualizing and characterizing PGCs migration in freshwater angelfish. This is the first research to utilize a heritable in vivo PGCs labeling system, enable precise tracking of PGCs migration in freshwater angelfish from early embryonic stage to the larva period using a fluorescent stereomicroscope.

### Unraveling PGCs migration pattern in freshwater angelfish

**Fig. 7:**
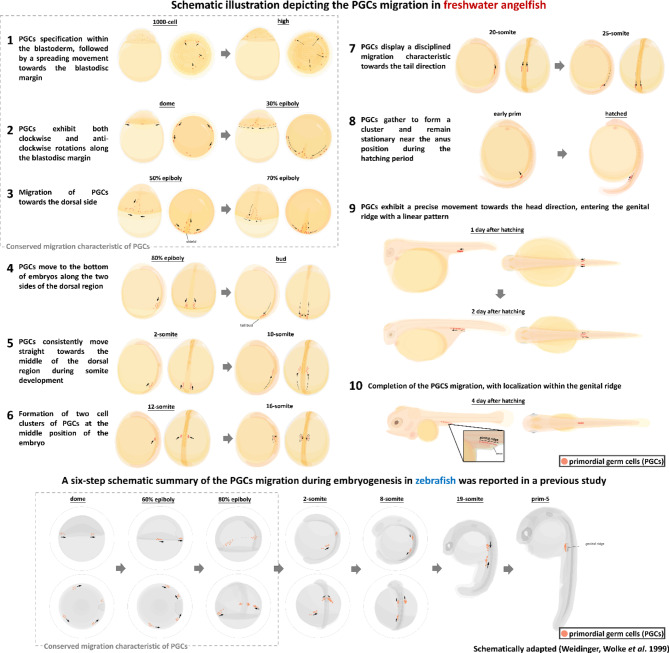
Schematic diagram comparing PGCs migration in freshwater angelfish and zebrafish. This study presents the first visualization and documentation of the complete PGCs migration process in freshwater angelfish, highlighting a 10-step migration pattern. Orange dots represent PGCs, and black arrows indicate the direction of migration. The schematic demonstrates unique characteristics of PGCs migration in freshwater angelfish, including a prolonged migration timeline and a multistage process involving linear movements across various embryonic stages. PGCs migration behavior in freshwater angelfish: (1) During early embryogenesis, PGCs are specified within the blastoderm, followed by spreading movements towards the blastodisc margin. (2) PGCs exhibit both clockwise and anti-clockwise rotations along the blastodisc margin during the early stages of epiboly development. (3) As epiboly progresses, PGCs migrate dorsally, gathering at the dorsal region near the embryonic shield. (4)−(6) During the segmentation period, PGCs migrate straight towards the dorsal midline, forming two distinct cell clusters at the midline of the embryo. (7)–(8) At later stages, PGCs exhibit disciplined migration toward the tailbud, eventually clustering near the anus region during hatching. (9)–(10) Hatching, PGCs migrate linearly towards the genital ridge, completing their localization within the genital ridge by 4 days after hatching. The schematic comparison highlights differences in PGCs migration between freshwater angelfish and zebrafish. Zebrafish PGCs follow a six-step migration process, characterized by quicker localization to the genital ridge (within 24 hpf). While some early rotational and midline alignment movements are conserved, the overall migration route and duration differ significantly between the two species. This comparison underscores the diversity in PGCs migration dynamics among teleosts, offering insights into species-specific reproductive biology. The prolonged migration period in freshwater angelfish provides a unique model for studying germ cell biology and its regulation.

Our findings reveal a detailed, multistage migration pattern of PGCs in freshwater angelfish (Supplementary Video). Notable characteristics of PGCs migration include spreading towards the blastodisc margin, peripheral rotation towards the dorsal sides, upward movement along the trunk-tail region from the tail bud to the dorsal midline, formation of cell clusters, orderly migration within the mesoderm towards the tail bud, and final alignment along the genital ridge above the intestinal tissue. Based on these observations, we summarized a 10-step process of PGCs migration (Fig. 7). Remarkably, PGCs migration in freshwater angelfish spans approximately 7 days (168 hpf), significantly longer than the 24 hpf required for complete localization in zebrafish. Similar prolonged migration periods have been reported in other teleosts, including eels (10 dpf), yellow catfish (7 dpf), pikeperch (at least 5 dpf), black rockfish, and Atlantic salmon^11,58–61^. While the periods of PGCs migration during embryonic development appear consistent among teleost, a wide variation in the migration patterns and routes has been reported. Current understanding, primarily derived from zebrafish, has made significant progress in unraveling the regulatory pathways and critical roles of specific genes governing PGCs migration. In zebrafish, PGCs exhibit rotated migration activity during the dome stage till early epiboly development, followed by lateral alignment, formation of two lateral clusters, and anterior migration for complete localization^49^. While the early stages of PGCs migration in freshwater angelfish resemble those in zebrafish, notable differences emerge during late epiboly. In angelfish, PGCs cluster at the bottom of the dorsal side, diverging from the zebrafish route. Diversity in PGCs migration patterns and durations among various organisms is extensively documented^4,62,63^. Factors such as cell motility, migration ability, polarization, and cell interactions influence PGCs migration. However, the molecular signaling mechanisms regulating these processes remain unexplored in freshwater angelfish. Future research should focus on understanding the regulation of guidance signals, identifying embryonic cell layers involved, and investigating the molecular and cellular mechanisms controlling motility, polarization, and signaling responses^64^. Understanding these regulatory mechanisms in freshwater angelfish may shed light on the nuanced aspects of PGCs migration. Compared to the chimeric-RNA labeling strategy, the transgenic approach provides greater stability and heritability, enabling efficient mass production of offspring for experimental purposes. The prolonged PGCs migration in freshwater angelfish also presents unique opportunities for in-depth experimental treatments and analyses.

### Variability in PGCs abundance and potential role in sexual dimorphism in freshwater angelfish

Our study revealed substantial individual variability in PGCs abundance, which appears to correlate with sexual dimorphism in freshwater angelfish. This variability raises intriguing questions about the molecular and cellular mechanisms underly differences in PGCs proliferation or cell division efficiency among individuals, highlighting the need for future studies to investigate these factors. Despite reproduction being a critical topic in aquaculture, few studies focus on PGCs in ornamental fish species. Understanding PGCs dynamics in ornamental species is essential, given their importance to the industry. For instance, maintaining balanced sexual dimorphism in broodstocks is critical for ensuring effective productivity^65^. Additionally, the discovery of PGCs creates opportunities for germ cell transplantation techniques, including surrogate production, fertility restoration, and minimizing inbreeding in aquaculture settings^66,67^. Combined with genome editing, a powerful tool for precision breeding, surrogate technology holds great potential for improving broodstocks in the ornamental fish industry^68,69^.

Our study further explores the role of PGCs as potential regulators of sexual dimorphism in freshwater angelfish. The discovery that higher PGCs counts correlate with a bias toward female sex development adds complexity to our understanding. These findings align with observations in zebrafish and medaka, where PGCs proliferation plays a critical role in promoting female development^15,70,71^. However, given the complexity and variability of sex development regulated by multiple factors, further research is essential to clarify these mechanisms in freshwater angelfish. In zebrafish, PGCs proliferation or meiosis is critical for promoting primary ovary-biased gene expression, contributing to female development^15^. Similarly, in medaka, PGCs have been shown to feminize somatic cells, supporting female gonad development^71^. Notably, our findings demonstrate variations in PGCs abundance among individuals, suggesting differences in PGCs proliferation or cell division efficiency. What distinguishes individuals with high or low PGCs proliferation activity? Current research suggests that energy availability, nutrients, and biosynthetic activity are required for cell proliferation^72^. Additionally, gene regulatory elements, transcription factors, and cell–cell communication signals play essential roles, as observed in cancer studies^73^.

Understanding the mechanisms driving PGCs proliferation may not only enhance our knowledge of cell biology but also provide valuable insights for practical applications. Future studies employing nutrient analysis and single-cell transcriptomics could elucidate the pathways linking PGC abundance to sex development, advancing both fundamental germ cell biology and its practical applications in aquaculture.

### Future research directions and conclusion

Our study has advanced the understanding of PGCs migration and it potential role in sex development in freshwater angelfish. Future research should explore the molecular and cellular mechanisms guiding PGCs migration, particularly the signals regulating migration stages and factors contributing to individual variability. Transcription factors such as NANOG, Oct-4, and SALL-4 have been identified as critical regulators of germ cell development in other species. As predicted TFBS were identified within the promoter elements of our expression construct, future studies employing mutagenesis or promoter assays could clarify the transcriptional regulating of the *ddx4* gene and germ cell specification in tilapia and related species. Comparative analyses of migration periods across teleost may also reveal evolutionary aspects.

Our findings establish reproductive biology, particularly within Cichlids and ornamental fish. Unique characteristics, such as adhesive eggs, parental care, aggressive behavior, and external sexual traits, further underscore its value. Despite limited research on angelfish, our study demonstrates its potential for advancing molecular biology and biotechnological applications, such as gene editing and germ cell manipulation. In conclusion, this study provides a foundational model for investigating germ cell biology and reproductive mechanisms in freshwater angelfish. By bridging existing knowledge gaps, we aim to contribute to reproductive biology and offer insights for practical applications in aquaculture and biotechnology.

## Electronic supplementary material

Below is the link to the electronic supplementary material.

Supplementary Material 1

Supplementary Material 2

## Data Availability

The sequence data generated during the current study are available in the GenBank of the National Center for Biotechnology Information (NCBI) repository. This includes DNA sequences of the 5′ UTR and promoter region of the transgenic expression construct, with accession number PQ031198, and the 3′ UTR of the transgenic expression construct, with Accession Number PQ031199.
