# Supplementary material for: Migration of primordial germ cells and their relationship of PGCs with sex development in transgenic germline-specific fluorescent freshwater angelfish (Pterophyllum scalare)
Source: Sci Rep. 2025 Jan 8;15:1308. doi: 10.1038/s41598-025-85480-7 (PMC11711190; doi:10.1038/s41598-025-85480-7)
Supplement: Supplementary file 1 — Supplementary Material 1 [file 41598_2025_85480_MOESM1_ESM.pdf]

Supplementary Figure 1

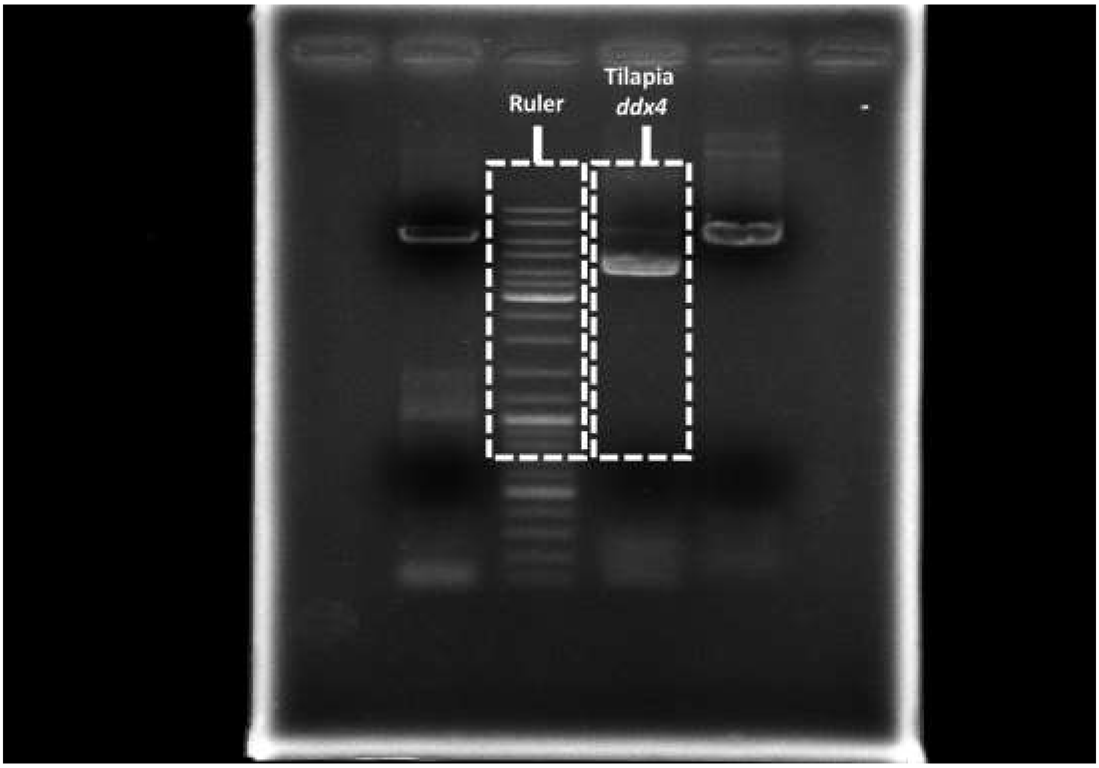

Original gels of Figure 1b. The panel shows gel electrophoresis analysis of a 4426 bp fragment amplified from the genome of Nile tilapia, including the promoter and 5'UTR region of the *ddx4* gene. Linear dotted lines indicate the cropped areas.

Supplementary Figure 2

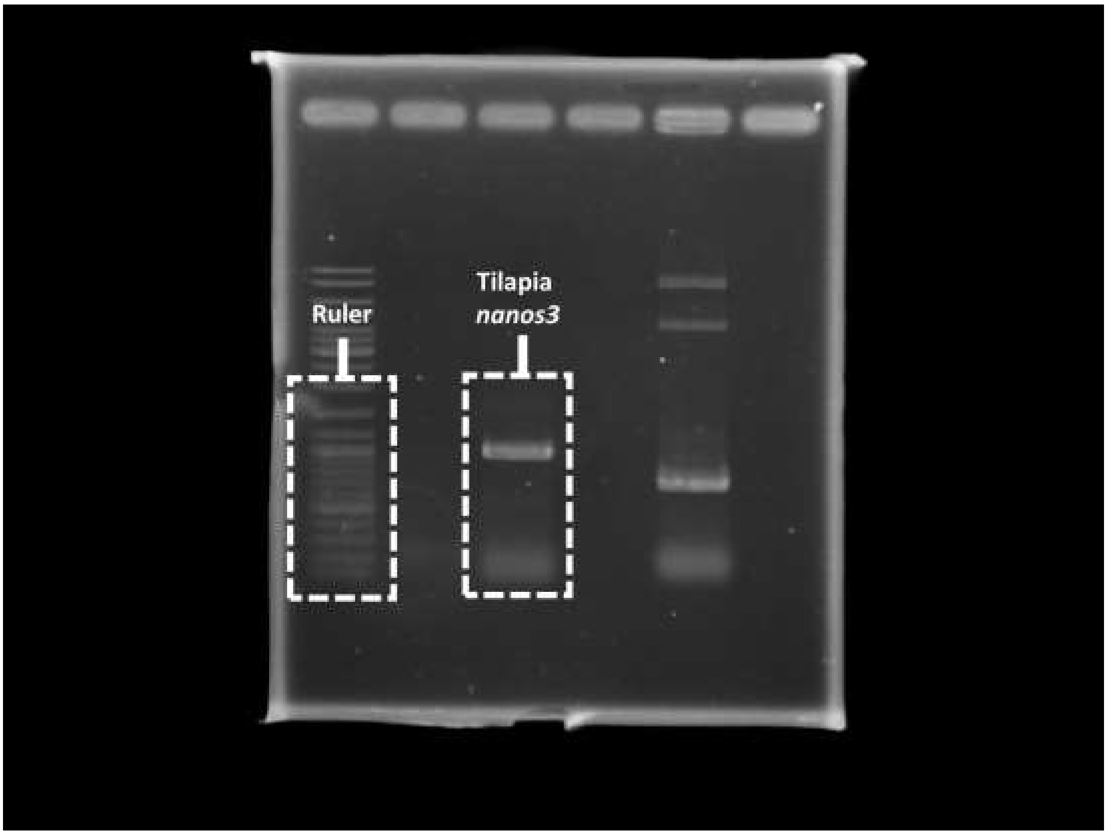

Original gels of Figure 1c. The panel shows gel electrophoresis analysis of a 1 kb fragment amplified from Nile tilapia, including the 3'UTR and flanking region of the *nanos3* gene. Linear dotted lines indicate the cropped areas.

Supplementary Figure 3

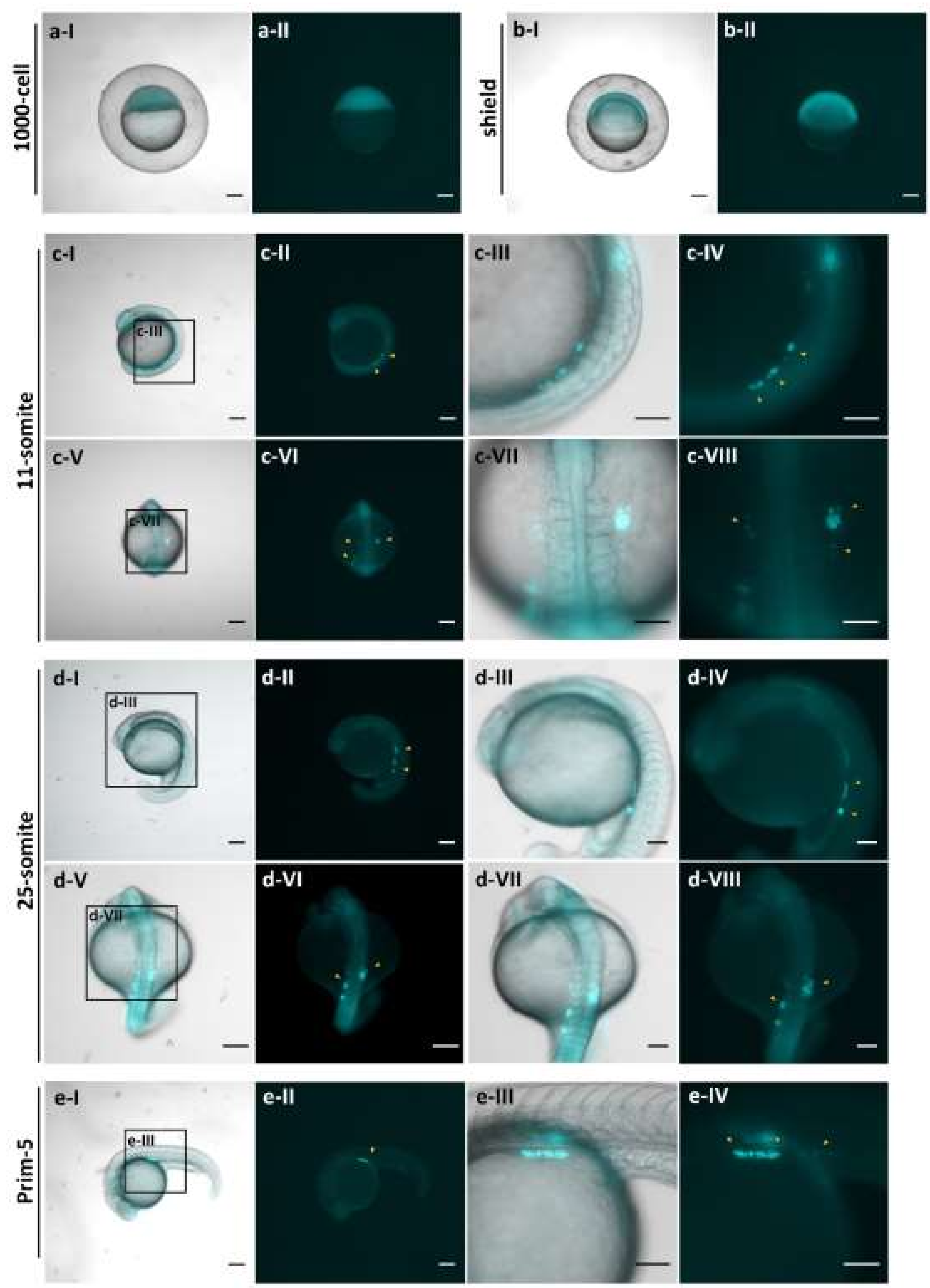

Evaluation of the PGCs labeling ability of the molecular construct in zebrafish.

F2 embryos of *Tg*(*ddx4:TcCFP13-nanos3*) zebrafish were collected, and the ability of

the molecular construct to label PGCs was visualized during embryonic development. Cyan fluorescent signal-positive cells were first observed during the somite development stage. Yellow arrows indicate PGCs in zebrafish. Micrographs of specific embryonic development stages, including (a) 1000-cell stage, (b) shield stage, (c) 11-somite stage, (d) 25-somite stage, and (e) prim-5 stage. Views include: (a)-(b) lateral view of the zebrafish embryo, (c-d) dorsal and side views of dechorionated zebrafish embryo (I-IV: side views, V-VIII: dorsal views), (e) side view of dechorionated zebrafish embryo. Bar: 200μm (e), 100μm (a-d).

Supplementary Figure 4

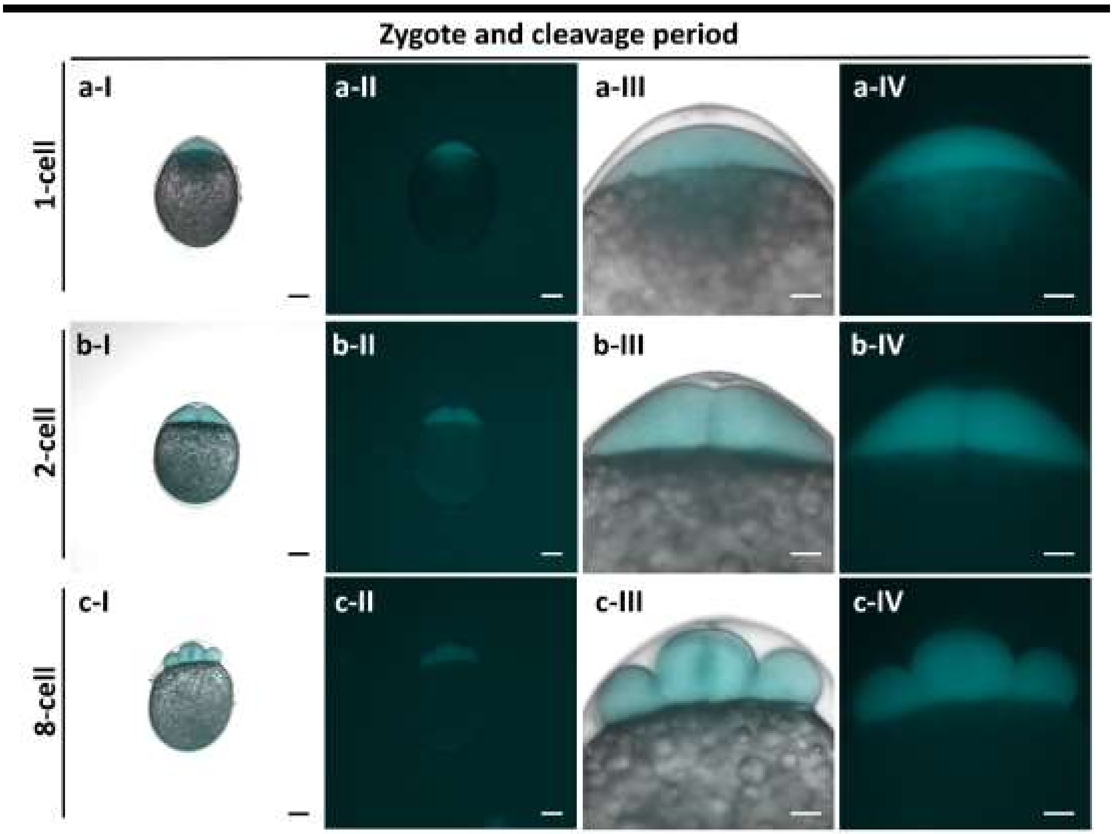

Micrographs visualizing PGCs migration in *Tg*(*ddx4:TcCFP13-nanos3*) transgenic freshwater angelfish embryo during the zygote and early cleavage period. Micrographs of embryonic development stages as follows: (a) 1-cell stage, (b) 2-cell stage, (c) 8-cell stage. No fluorescent signals are observed during these stages. Bar: 200μm (a)-(c) I-II, 100μm (a)-(c) III-IV.

Supplementary Video

Schematic animation of PGCs migration in freshwater angelfish. This video illustrates the migration process of PGCs in transgenic freshwater angelfish. The animation highlights key stages of PGCs migration, starting from the early embryonic stages and progressing through to the larval period. The PGCs are represented as orange dots, and the direction of their migration is indicated by black arrows. The video captures the multistage and complex migration route, including the initial spreading towards the blastodisc margin, rotation towards the dorsal region, clustering along the trunk-tail region, and final localization at the genital ridge above the intestine. This schematic visualization provides an overview of the PGCs migration process in freshwater angelfish, offering a clearer understanding of the dynamic movement and localization patterns observed in this species.
